# A Rare Syndrome Causing Neurogenic Dysphagia

**DOI:** 10.1007/s00455-020-10208-w

**Published:** 2020-11-04

**Authors:** Antonio Zito, Paola Bini, Massimiliano Todisco, Giuseppe Cosentino, Simone Mauramati, Matteo Paoletti, Vincenzo Marasco, Enrico Marchioni, Enrico Alfonsi

**Affiliations:** 1 Department of Brain and Behavioral Sciences, University of Pavia, Pavia, Italy; 2 Neuroncology and Neuroinflammation Unit, IRCCS Mondino Foundation, Pavia, Italy; 3 Clinical Neurophysiology Unit, IRCCS Mondino Foundation, Via Mondino 2, 27100 Pavia, Italy; 4 Department of Otorhinolaryngology, University of Pavia, IRCCS Policlinico San Matteo Foundation, Pavia, Italy; 5 Neuroradiology Unit, IRCCS Mondino Foundation, Pavia, Italy; 6 Università degli Studi di Milano, Milan, Italy

## History

A 46-year-old right-handed man with a medical history of refractory stage IIIB, ALK-negative, anaplastic large-cell lymphoma came to our attention. He had previously been treated with several chemotherapy drugs, including brentuximab vedotin. Two weeks after completing salvage chemotherapy, the patient presented with progressive swallowing and speech difficulties. At this stage, he was admitted to our hospital for further diagnostic work-up and clinical management. Neurological examination showed severe dysarthria and bilateral weakness of facial, oromandibular and palatal voluntary movements. The patient could barely open his mouth and protrude his tongue on command. However, reflex movements (e.g., blink and gag reflexes) and activation of emotional and automatic motor responses (e.g., facial response to painful stimulus, laughing at jokes, mouth opening during yawning) were broadly spared.

**Fig. 1:**
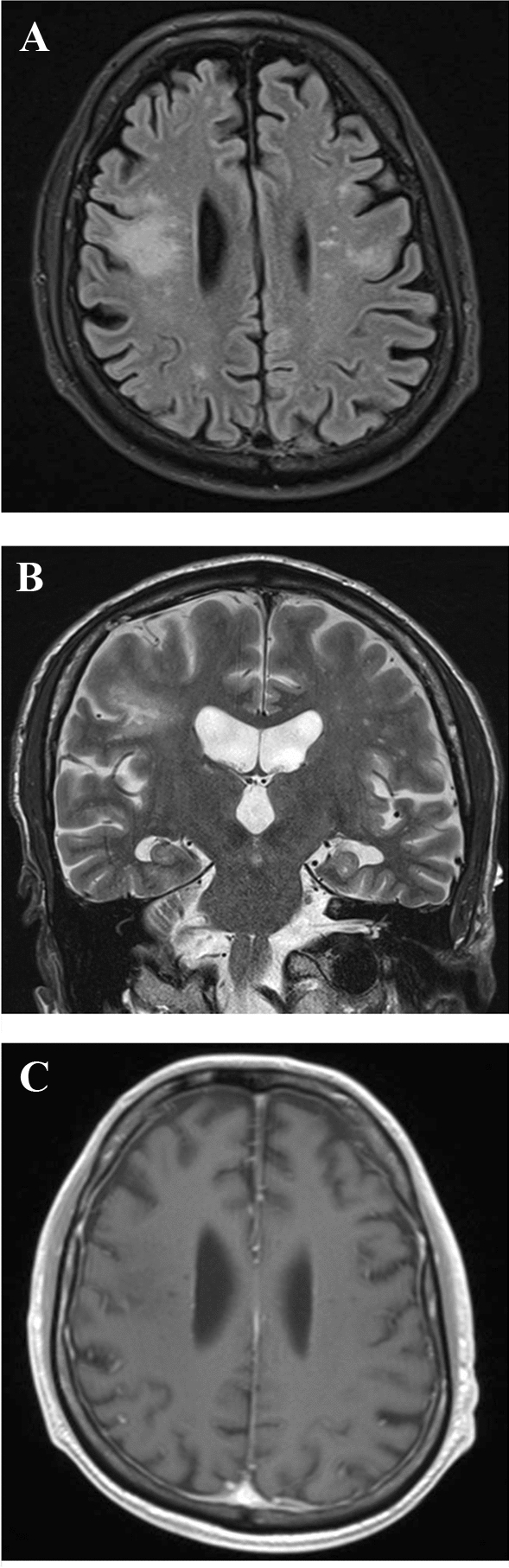
Brain magnetic resonance imaging of the patient. Bilateral hyperintense alterations of the subcortical white matter of the frontal operculum, mostly on the right side, on axial FLAIR (**a**) and coronal T2-weighted sequences (**b**). The lesions are hypointense and do not show contrast enhancement on axial T1-weighted sequences (**c**). The Milky Way sign is evident on both T1- and T2-weighted sequences

Blood tests showed severe lymphocytopenia (77/mm^3^). Brain magnetic resonance imaging displayed T1-hypointense, T2-hyperintense abnormalities of the frontal subcortical white matter, also involving the U-fibers and the frontal operculum bilaterally (mainly on the right side). No mass effect or contrast enhancement after gadolinium administration was evident (Fig. 1). On both T1- and T2-weighted sequences, the Milky Way sign was also observed (i.e., multiple point-like alterations surrounding the main lesions) (Fig. 1). Magnetic resonance spectroscopy (at intermediate echo time of 135 ms) of the right opercular lesion showed increased choline and decreased N-acetyl aspartate levels, and a lactate peak, indicating the presence of neuronal loss, necrosis and increased cell turnover. Cerebrospinal fluid (CSF) analysis showed a normal cell count, normal albumin quotient, and absence of oligoclonal banding, but the polymerase chain reaction was positive for JC virus DNA (390 copies/mL).

**Fig. 2:**
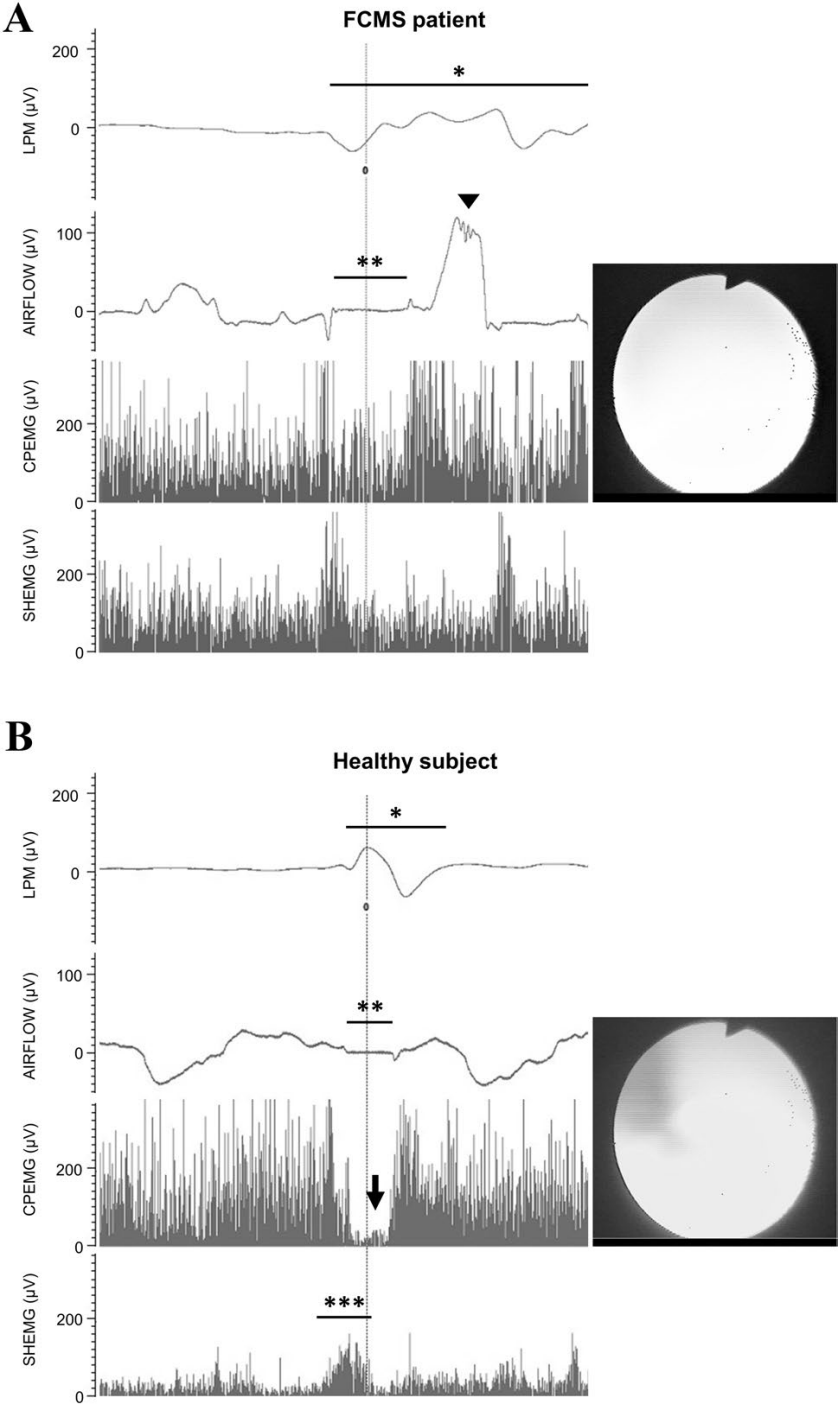
Electrokinesiographic/electromyographic study of swallowing in our FCMS patient (**a**) and a representative trace from a healthy subject (**b**). The first channel records the LPM using a piezoelectric transducer applied to the skin over the cricothyroid membrane (*LPM during the pharyngeal phase). The second channel records breathing activity by means of a nasal cannula connected to a piezoelectric transducer (negative and positive deflections represent inspiration and expiration, respectively) (**swallowing apnea during the pharyngeal phase, while the arrowhead shows the post-swallowing inspiration in the FCMS patient). The third channel records the CPEMG with a percutaneous concentric needle (the arrow indicates the pause of the CPEMG during the pharyngeal phase, physiologically present in the healthy subject and absent in the FCMS patient). The fourth channel records the SHEMG using surface electrodes (***SHEMG triggering an effective swallowing in the healthy subject). The vertical line represents the occurrence of the “white-out” on simultaneously performed FEES (shown on the right panel). *CPEMG* electromyographic activity of the cricopharyngeal muscle, *FCMS* Foix–Chavany–Marie syndrome, *FEES* fiberoptic endoscopic evaluation of swallowing, *LPM* laryngopharyngeal mechanogram, *SHEMG* electromyographic activity of the suprahyoid/submental muscles

To allow more in-depth exploration of the features of neurogenic dysphagia in this patient, he underwent an electrokinesiographic/electromyographic study of swallowing (EES), which was performed simultaneously with a fiberoptic endoscopic evaluation of swallowing (FEES). EES was carried out according to a previously described protocol [1]. Breathing activity was also assessed, by means of a nasal cannula connected to a piezoelectric transducer. The investigation was performed with small (3–6 mL) volumes of water (colored with methylene blue) and jelly, administered directly into the oral cavity using a disposable syringe. The oral preparatory and propulsive phases of swallowing were both absent. The patient was indeed unable to hold the bolus in his oral cavity and to propel it against his palate with his tongue, regardless of the bolus consistency (i.e., water or jelly). The bolus thus reached the pharynx through passive drop (“posterior spillage”). EES also showed repetitive and disorganized activity of the suprahyoid/submental muscles and no triggering of effective swallowing, a pattern consistent with oral-buccal-lingual apraxia (Fig. 2a). Therefore, the pharyngeal phase of swallowing was activated only involuntarily and incompletely. After this activation of the pharyngeal phase by passive bolus drop, the onset timing both of the laryngopharyngeal mechanogram (LPM) and of swallowing apnea was within normal values (Fig. 2a). However, EES showed prolonged duration of both LPM and swallowing apnea, followed by a pathological post-swallowing inspiration. Absent relaxation of the cricopharyngeal muscle, which is the main portion of the upper esophageal sphincter, was also observed (Fig. 2a). After the “white-out”, FEES showed bolus retention in the valleculae and piriform sinuses, and airway penetration and aspiration with both bolus consistencies. Reflexive cough was weak and ineffective.

This patient’s severe dysphagia required nasogastric tube feeding and a percutaneous endoscopic gastrostomy was planned.

## Diagnosis

In view of the patient’s clinical features, combined with the localization of lesions on neuroimaging and the CSF findings, Foix–Chavany–Marie syndrome (FCMS) related to progressive multifocal leukoencephalopathy (PML) was diagnosed.

FCMS, also known as opercular syndrome, is a rare neurological entity characterized by voluntary palsy, but preserved reflexive and automatic motor functions of the trigeminal, facial, glossopharyngeal, vagus and hypoglossal nerves. This syndrome is caused by bilateral lesions of the frontal operculum, classically affecting voluntary control of the facial, oromandibular, pharyngeal and laryngeal muscles. The presence of divergent corticobulbar pathways for voluntary and automatic or emotional motor activation of craniofacial muscles is the anatomical basis of FCMS symptoms. For example, opercular neurons, which are responsible for voluntary movements, send supranuclear fibers to the nuclei of the cranial nerves, whereas the automatic or emotional activation of craniofacial muscles depends on another pathway, from the amygdala and hippocampus to the brainstem.

## Discussion

The swallowing abnormalities detected in the case here reported are not consistent with the conventional concept of automatic-voluntary movement dissociation in FCMS. We observed not only an impairment of the oral preparatory and propulsive phases but also involvement of the automatic-reflexive mechanisms of the pharyngeal phase of swallowing (i.e., prolonged duration of the LPM and absent relaxation of the cricopharyngeal muscle). The oral phase alterations can be considered unsurprising, being related to ineffective voluntary control of corticobulbar pathways from the opercular cortex. The abnormalities of the pharyngeal phase, on the other hand, were unexpected, given that this phase is classically considered to depend on involuntary activation of the swallowing central pattern generator, which is located in the medulla oblongata. Nevertheless, our findings are in keeping with the role played by the frontal operculum in the control and modulation of the automatic-reflexive mechanisms of swallowing [2]. Moreover, in our patient, the greater involvement of the right (non-dominant) hemisphere could explain an additional impairment of the pharyngeal phase of swallowing. In fact, it has been suggested that the non-dominant hemisphere is particularly engaged in involuntary swallowing activities, in contrast with the dominant hemisphere control of voluntary mechanisms [3].

Loss of suprasegmental control from the right opercular cortex may also lead to abnormal respiratory-swallowing coordination (i.e., prolonged duration of the swallowing apnea followed by pathological post-swallowing inspiration), increasing the risk of airway penetration and aspiration. Indeed, breathing is modulated by cortical structures (including the frontal operculum, insula and orbitofrontal cortex) projecting to limbic areas and connected with medullary cardiorespiratory regulatory centers. This pathway is at least partly shared with networks involved in the swallowing function, and therefore represents a neuronal substrate for respiratory-swallowing coordination [4].

In addition, we hypothesize that the preservation of pharyngeal afferent sensory inputs and of brainstem structures may account for the normal onset timing of LPM and of swallowing apnea observed in this patient.

Dysphagia related to FCMS is an uncommon symptom in PML, to our knowledge reported only once in the literature to date [5]. In our case, the PML cortical lesions proved to be a useful model of neurogenic dysphagia, providing new pathophysiological insights into swallowing abnormalities in FCMS.

## References

[1] Alfonsi E, Restivo DA, Cosentino G, De Icco R, Bertino G, Schindler A, Todisco M, Fresia M, Cortese A, Prunetti P, Ramusino MC, Moglia A, Sandrini G, Tassorelli C (2017). Botulinum toxin is effective in the management of neurogenic dysphagia. Clinical-electrophysiological findings and tips on safety in different neurological disorders. Front Pharmacol.

[2] Martin RE, Goodyear BG, Gati JS, Menon RS (2001). Cerebral cortical representation of automatic and volitional swallowing in humans. J Neurophysiol.

[3] Daniels SF, Corey DM, Fraychinaud A, DePolo A, Foundas AL (2006). Swallowing lateralization: the effects of modified dual-task interference. Dysphagia.

[4] Herrero JL, Khuvis S, Yeagle E, Cerf M, Mehta AD (2018). Breathing above the brain stem: volitional control and attentional modulation in humans. J Neurophysiol.

[5] Dijkstra F, Guldolf K, Schotsmans K, Maréchal E, Hernalsteen D, Crols R (2018). Foix-Chavany-Marie syndrome as the presenting sign of HIV-related PML. Neurol Clin Pract.

